# A toolkit for assembly of targeting clones for *C. elegans* transgenesis

**DOI:** 10.17912/micropub.biology.000966

**Published:** 2023-10-02

**Authors:** Emma Knoebel, Scott Dour, Michael Nonet

**Affiliations:** 1 Department of Neuroscience, Washington University Medical School, St. Louis, MO, USA; 2 Benson Hill, St. Louis, MO, USA

## Abstract

Transgenic worms are a key resource for *C. elegans* researchers dissecting molecular pathways using this simple metazoan model system. Transgenes provide an avenue to visualize developmental events, cellular processes as well as real-time signal events in live animals using genetically encoded sensors. Generation of these tools has become increasingly efficient with the advent of numerous integration methods including transposon, CRISPR and recombinase-mediated integration. A growing limitation in transgene production is the assembly of the targeting constructs used to direct insertion of sequences into the genome. Here we present a toolkit that facilitates rapid assembly of complex reporters using a Golden Gate (GG) cloning-based approach. Co-assembly of one to eight DNA segments into an integration vector can be routinely obtained at high efficiency using a library of entry plasmids. The toolkit consists of 20 *SapI* GG entry vectors and 100 *SapI* GG insert plasmids containing a variety of promoters, FPs, tags, linkers, ORFs, 3' UTRs and numerous components for bipartite expression systems that can be mixed to create a huge repertoire of reporter constructs. The assembly process also works well with PCR products and 5' phosphorylated double stranded oligonucleotides, and such DNAs can be used to supply novel genes, promoters, and tags into the pipeline. In addition, the toolkit also provides a series of 12 empty *BsaI* -based GG assembly vectors that facilitate the construction of additional *SapI* GG plasmids containing novel inserts. A manual outlining the entire approach is provided as an appendix as well as a Microsoft® Excel based assembly tool which allows the user to choose individual inserts among the libraries of clones at each position in the assembly template and output an annotated sequence. The assembly process can easily be multiplexed and is typically over 90% efficient. The approach is sufficiently efficient to make microinjection rather than clone generation the limiting factor in transgene generation.

**Figure 1. Overview of Golden Gate (GG)-based targeting plasmid assembly:**
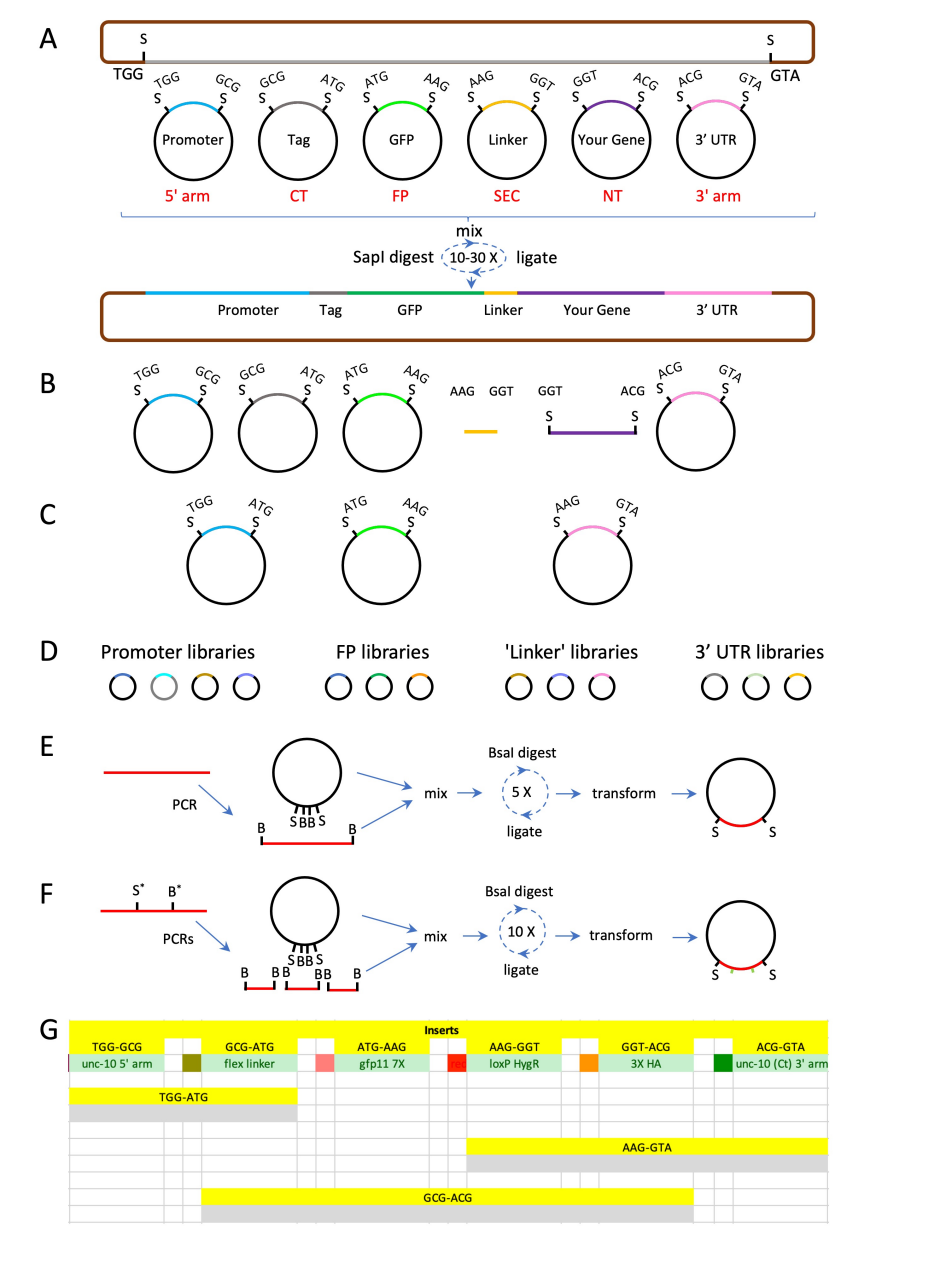
**A)** An RMCE vector and six insert-containing entry clones, each with distinct *SapI* generated 3 bp overlaps, are co-assembled using a *SapI* GG ligation protocol. The entry vector backbones are KanR (black) while the backbone of the targeting vector and final clone are AmpR (brown). In red is the designation given to each 'slot' with distinct *SapI* overhangs. **B)** PCR products (purple), hybridized oligos (yellow) and plasmids can be combined in different combinations to create targeting plasmids of varying complexity. **C)** For creating simpler constructs, entry clones which combine slots are used. **D** ) Clone libraries of promoters, fluorescent proteins, linkers, and 3' UTRs provide a wealth of reagents for assembling new constructs. **E-F)** A variety of KanR vectors facilitates creating novel insert clones using efficient BsaI GG-based assembly strategies. **E)** A *BsaI* GG strategy can be used to create novel entry clones by amplifying the insert with oligonucleotides containing *BsaI* sites and performing a GG reaction into KanR vectors. **F)** *BsaI* and *SapI* sites in inserts can be removed using a *BsaI* GG strategy that lesions each site in the insert. **G)** Screen capture of a portion of the Excel-based 'GG plasmid builder' assembly tool which facilitates visualization of construct design options and testing of designs *in silico* . S, *SapI* sites; B, *BsaI* sites. Plasmids and DNA fragments are not drawn to scale.

## Description

Several efficient methods for creating *C. elegans* transgenic animals have been developed over the last decade including transposon, CRISPR double stranded break repair and site-specific recombinase-based methods (reviewed in Nance and Frøkjær-Jensen, 2019). However, assembly of targeting clones for these techniques remains a significant constraint in developing novel lines. Golden Gate (GG) cloning (Engler et al., 2008) is a single tube plasmid assembly method based on type II restriction endonucleases, such as *SapI* or *BsaI* , that cleave DNA adjacent to, but outside of a specific non-palindromic sequence. Digestion with these enzymes yields products with 2 to 4 bp overhangs that are not constrained by the recognition sequence. Careful design of molecular reagents permits the precise co-assembly of multiple DNA fragments with each fragment containing distinct overhangs and all fragments lacking the enzyme recognition site. Using such reagents one can mix a plasmid backbone and multiple (up to 20) inserts and perform a repetitive digestion-ligation reaction ( **Figure 1A** ). In addition to plasmids, hybridized oligonucleotides pairs and PCR products can also be used as inputs in the reactions ( **Figure 1B** ). GG reactions are exceedingly efficient because, while all input plasmids are repeatedly cleaved, the final product does not contain the enzyme cleavage site and is the only circular plasmid product produced in the reaction. To efficiently generate targeting plasmids for CRISPR-mediated integration Schwartz and Jorgensen (2016) first used this approach naming it 'SapTrap' and it was subsequently extended by Dickinson et. al. (2018). In the Dickinson approach, 6 entry slots each containing distinct, but overlapping, 3 bp overhangs are arranged in a specific order and called the '5' arm', 'CT', 'FP', 'SEC', 'NT', and '3' arm' slots ( **Figure 1A** ). In their version, the 5' arm and 3' arm slots are devoted to introducing the two homology arms for driving recombination. The insert is assembled from the 4 remaining slots. Typically, the CT slot encodes a linker or tag, the FP slot encodes the fluorescent protein, the SEC slot encodes a self-excisable *hygR* *sqt-1 hsp-16p* CRE selection cassette, and the NT slot encodes a linker or tag. In the Schwartz SapTrap approach the FP and SEC slots are combined and called the 'Tag and Selectable Marker' slot.

I co-opted this system for the assembly of RMCE targeting plasmids (Nonet, 2020; Nonet, 2023) which differ significantly from CRISPR targeting plasmids because both the selection cassette and the FRT recombination sites are encoded in the vector backbone rather than as inserts. This opens the 5' arm, SEC and 3' arm slots to encode components of the insert permitting the assembly of more 'complex' clones ( **Figure 1A** ). For RMCE, the 5' arm slot often encodes a promoter and the CT slot encodes a tag. In some cases, these two slots are combined into a 5' arm-CT slot that encodes a promoter without a tag. For creating N-terminal protein fusions, the FP slot encodes a protein tag of interest (e.g., an FP, N-terminal tag epitope tag, a BioID tag, etc.), the SEC slot encodes a short peptide linker, the NT slot encoded the protein encoding gene of interest, and the 3' arm slot encodes a 3' UTR ( **Figure 1A** ). For C-terminal fusions, the role of the NT and FP slots is swapped. Slot skipping insert clones are used to create simpler clones ( **Figure 1C** ). For example, often the goal is to express a protein (e.g., FP) under the control of a promoter. In these cases, the promoter is encoded in a 5' arm-CT slot, the FP in the FP slot, and the 3' UTR in a SEC-3' arm slot. Over the past few years, we have assembled and tested many slot clones including > 20 promoter clones, many FP clones (in both the FP and NT slots), numerous 3' UTR clones and a variety of linkers, tags, splice leaders and other miscellaneous inserts ( **Figure 1D** ).

The existing set of clones can be used to create novel clones by combining existing clones in other orders. To create a clone, one mixes equimolar concentrations of all plasmids (or linear inserts) and the vector and performs a repetitive ligation-digest reaction, then transforms the reaction into *E. coli* . For example, a pharyngeal gland cell-specific clone to label transport vesicles in a blue fluorescence channel could be assembled by combining the *phat-4* promoter, *BFP* , a flexible linker, *rab-3* , and a *tbb-2* 3' UTR. Since these clones pre-exist, the targeting construct could be assembled via a GG reaction performed in the morning, an *E. coli* transformation in the afternoon, followed by isolation of DNA of the novel clone late in the next afternoon. GG assembly is efficient enough that one can multiplex the reactions by combining partial molar quantities of multiple different clones of an individual slot (such as 4 different promoters) to create several related clones from one reaction. The system is robust enough that even relatively novice lab personnel in our lab regularly obtain GG derived clones at a frequency of > 90% of colonies isolated. However, it is still imperative that clones be analyzed before use.

In the creation of integration clones that introduce novel sequences one has two choices. Our approach has been to create intermediate entry clones to develop libraries of reagents, then use those reagents to create the integration plasmids ( **Figure 1D** ). However, an alternative strategy that we occasionally use is to amplify the sequence of interest for a 'slot' by flanking the PCR product with *SapI* sites and using the purified PCR product directly. The advantage of using the PCR product is saving the time and effort required to create and characterize the novel entry clone. The two disadvantages of using PCR products are 1) that PCR products which are contaminated with primer dimer products perform poorly in GG reactions and 2) that the resulting plasmids are more likely to contain errors and thus are best sequenced to confirm the integrity of the plasmid. Creating an intermediate clone can be time saving in the long run if it is likely that one will use the same sequence to create multiple different integration plasmids. In addition, it greatly facilitates reagent sharing.

To facilitate creation of entry clones we developed a series of Kan ^R^ vectors with strategically positioned *BsaI* sites and *SapI* sites that allow one to create *SapI* entry clones using a *BsaI* GG reaction. These vectors have been created for each single slot and for some common combined slots. For inserts that do not contain either a *SapI* or *BsaI* site, assembly of a new entry vector is rapid and efficient, requiring one to only perform a PCR reaction using oligonucleotides containing the appropriate 5' *BsaI* sites followed by a quick GG reaction with the appropriate vector ( **Figure 1E** ). The most significant general limitation of the *SapI* GG approach is that inserts cannot contain *SapI* sites. For inserts that do contain *SapI* sites, the sites can be removed by mutagenesis. Multiple approaches including Gibson assembly can be used to perform this task. The approach we often use is to use a *BsaI* GG reaction lesioning each *SapI* site (and *BsaI* site, if present) in the insert, appending *BsaI* sites on the end of the fragments, then co-assembling the products with a *BsaI* Golden Gate reaction ( **Figure 1F** ). Detailed descriptions of both the Gibson assembly and *BsaI* approaches are described in an accompanying manual ( **Extended data GG Manual** ). In cases where multiple *SapI* or *BsaI* sites are present, synthesis of a synthetic DNA that lesions these sites is often the most expedient approach. While expending the effort to remove all *BsaI* sites from inserts may seem overzealous and inefficient, the approach has permitted us to assemble a large series of *BsaI* lacking entry clones and targeting vectors for rRMCE. These serve as templates for the assembly of novel entry clones and vectors including ones with major re-organization of components (Nonet, 2023) using efficient GG *BsaI* reactions.

The toolkit provides reagents to build many different types of targeting vectors. Many of the reagents developed by Schwartz and Dickinson are compatible with this approach and vice versa and thus also extend the tool kit for that well vetted approach to transgenesis. In addition, the kit contains the core components for building GAL4, Tet, QF, and LexA driver and reporter bipartite constructs (Wei et al., 2012; Wang et al., 2017; Mao et al., 2019; Nonet, 2020; Nonet, 2021) . Furthermore, most of the reagents described in this work are also compatible with creating CRISPR targeting plasmids. In particular, we include reagents for creating targeting plasmids that contain a floxed *hygR* selection cassette without a source of Cre. The plasmids can then be integrated using DNA or ribonuclear complex-based CRISPR approaches (Nance and Frøkjær-Jensen, 2019) using hygromycin selection followed by excising the selection cassette by outcross to a germline Cre expressing line (Nonet, 2023) . In this strategy, the targeting sequences are inserted into a plasmid containing an FRT site in the backbone and the injections are performed in a strain expressing germline FLP to prevent array formation, which greatly reduces the background of non-integrated hygR progeny. FLP-mediated breakdown of the array may also increase availability of targeting sequences for integration. A more detailed manuscript describing this approach is forthcoming.

A detailed list of all plasmid reagents described in this paper including links to annotated sequence files in genbank format is provided in **Extended data Table 1** . *In silico* assembly of novel constructs can easily be accomplished using ApE (Davis and Jorgensen, 2022) , a free DNA analysis and manipulation program. ApE provides a Golden Gate assembly tool which creates an annotated genbank file of the product plasmid from input sequences. However, ApE does not provide a simple way to visualize all the potential clones that can be created using the various entry clones. To enable easier visualization of the possibilities we constructed a Microsoft® Excel based assembly tool ( **Figure 1G, Extended data GG Assembly Builder V** ) that contains all reagents described herein and uses pulldown menus to permit the user to build plasmids *in silico* yielding both an unannotated and an annotated sequence from the combination of reagents selected. Finally, we also provide a manual ( **Extended data GG manual** ) which provides detailed protocols for the different approaches used in the Nonet lab to assemble targeting plasmids using Golden Gate assembly and build novel entry clones using traditional, Golden Gate and Gibson assembly approaches.

## Methods

Plasmid and vector constructions

The term 'vector', rather than 'plasmid', is used to distinguish the parental integration vectors from integration plasmids that contain specific sequences inserted in the MCS of the vectors. All PCR amplifications for plasmid and vector constructions were performed using Q5 polymerase (New England Biolabs, Ipswich, MA). Most PCR reactions were performed using the following conditions: 98°C for 30 sec, followed by 30 cycles of 98°C for 10 sec, 62°C for 30 sec, 72°C for 1 min/kb). PCR products were digested with *DpnI* to remove template if amplified from a plasmid, then purified using a standard Monarch (New England Biolabs) column purification procedure. Restriction enzymes (except for *LguI* ), T4 DNA ligase, and polynucleotide kinase were purchased from New England Biolabs. Golden Gate (GG) reactions (Engler et al., 2008) were performed as described in Nonet (2020) except that in some cases *LguI* (Thermo Scientific™, Waltam, MA) was used in place of *SapI* . The *E. coli* strain DH5α was used for all transformations. Sanger sequencing was performed by GENEWIZ (South Plainfield, NJ) and nanopore sequencing by Plasmidsaurus (Eugene, OR). Oligonucleotides were obtained from MilliporeSigma (Burlington, MA) and synthetic DNA fragment were purchased from Twist Biosciences (South San Francisco, CA). A detailed description of all constructs is provided in **Extended data Methods** . A compilation of reagents including the sequence of all plasmids, vectors, oligonucleotides, and synthetic fragments used in this study is provided in **Extended data Table 1** .

## Reagents

A complete list of available reagents is listed in **Extended data Table 1** . The reagents are organized into a 24 plasmid starter kit, a 96 plasmid expansion kit, and a 12 plasmid CRISPR-specific kit. These three kits are available by request from the Nonet lab. If interest warrants it, the plasmids will be made available through Addgene. Also provided as extended material are a manual detailing the protocols and an Excel-based GG assembly builder. Additional information on the method is available on the Nonet lab website at https://sites.wustl.edu/nonetlab/genome-manipulation/.

## Extended Data

Description: Detailed description of construction of >100 plasmids described in this study. Resource Type: Text. DOI: 10.22002/2kjkx-vwx32

Description: Excel file listing the plasmids available, their sequence, and links to annotated files. Resource Type: Text. DOI: 10.22002/34va1-qkd68

Description: Excel 'program' for assembling GG clones. Resource Type: InteractiveResource. DOI: 10.22002/3nkgg-4zd92

Description: Manual describing detailed protocols and work flow for assembling clones. Resource Type: Text. DOI: 10.22002/r6bc7-zpm23
